# Study on Physical Properties of Mortar for Section Restoration Using Calcium Nitrite and CO_2_ Nano-Bubble Water

**DOI:** 10.3390/ma13173897

**Published:** 2020-09-03

**Authors:** Ho-jin Kim, Hyeonggil Choi, Heesup Choi, Bokyeong Lee, Dongwoo Lee, Dong-Eun Lee

**Affiliations:** 1 Daegu Gyeongbuk Branch, Korea Testing & Research Institute, Daegu 41516, Korea; white002@ktr.or.kr; 2 School of Architecture, Civil Environment and Energy Engineering, Kyungpook National University, Daegu 41566, Korea; dolee@knu.ac.kr; 3 Department of Civil and Environmental Engineering, Kitami Institute of Technology, Hokkaido 090-8507, Japan; 4 Intelligent Construction Automation Center, Kyungpook National University, Daegu 41566, Korea; bklee11@knu.ac.kr; 5 BUKUK (Neutralization & Prevention of Structure Hazard System), Gyeonggi-do 17001, Korea; hit6585@gmail.com

**Keywords:** calcium nitrite, CO_2_ nanobubble water, section restoration, mortar

## Abstract

This study investigated the physical properties of section-restoration mortar with calcium nitrite (Ca(NO_2_)_2_) and carbon dioxide (CO_2_) nanobubble mixing water to develop materials and methods for the repair and reinforcement of cracks in reinforced concrete (RC) structures. As the calcium nitrite content increased, the generation rate and generated amount of nitrite-based hydration products also increased, owing to the rapid reaction between NO_2_^−^ ions in calcium nitrite and C_3_A(Al_2_O_3_). Further, the reaction with C_3_S and C_2_S was accelerated, thereby increasing the generation rates of Ca(OH)_2_ and C-S-H. The large amount of Ca^2+^ ions in these hydration products reacted with CO_3_^2−^ ions in CO_2_ nanobubble water, thereby increasing the generation of calcite-based CaCO_3_ in the cement matrix. This appears to have affected strength development and durability improvement via the densification of the structure. These results suggest that the performance of polymer cement mortar for repairing concrete structures can be improved if calcium nitrite and CO_2_ nanobubble water are properly combined and applied.

## 1. Introduction

In recent years, global efforts have focused on reducing industrial carbon dioxide (CO_2_) emissions. In this light, South Korea has aimed to reduce its industrial CO_2_ emissions. The construction industry accounts for approximately 40% of total industrial CO_2_ emissions [1]. Further, cement production and concrete manufacturing account for approximately 5% of these emissions. Therefore, it is essential to reduce CO_2_ emissions in the construction industry. One way of doing so is to properly maintain the existing structures through repair and reinforcement instead of building new structures. For this purpose, repair and reinforcement methods with reduced environmental loads must be developed for extending the service life of concrete structures.

Concrete is widely used in the construction industry. The tensile strength of concrete is significantly lower than its compressive strength. In particular, cracks inevitably occur in buildings owing to various problems in the mix design and construction process [2]. Microcracks in buildings are generally not considered significant structural problems [3]. Nonetheless, microcracks facilitate the penetration of chlorides and CO_2_ that are highly likely to critically degrade the structural stability of reinforced concrete (RC) structures [4]. Cracks in RC structures also negatively impact the appearance of these structures [5]. Therefore, crack formation must be prevented. Further, materials and methods for crack repair and reinforcement must be developed to realize high-performance RC structures and sustainable buildings.

A previous study noted that in a moist environment, concrete containing small cracks showed a self-healing phenomenon in which part of the crack was filled [6,7,8,9,10,11,12,13,14]. This phenomenon occurred due to the rehydration of cement particles and the precipitation of CaCO_3_; specifically, CaCO_3_ was generated by the reaction between Ca^2+^ in concrete and CO_3_^2−^ dissolved in water. Further, a strong self-healing effect was observed when CO_2_ nanobubble water was used [2]. Calcium nitrite (Ca(NO_2_)_2_) is widely used as the main component in antifreeze admixtures. Increasing the calcium nitrite content accelerates the hydration reaction of C_3_A and C_3_S (both of which are components of cement) and thereby increases the generation of ettringite-based hydrate (Aft). Further, the generation of nitrite-based hydrates in large quantities owing to the reaction between C_3_A(Al_2_O_3_) and NO_2_^−^ increases the initial strength of concrete [15].

In this light, the present study investigates the physical properties of section-restoration mortar with calcium nitrite and CO_2_ nanobubble mixing water to develop materials and methods for the repair and reinforcement of cracks that occur in RC structures.

## 2. Materials and Methods

### 2.1. Experimental Plan

Table 1 and Table 2 show the experimental plan and mortar mix proportion of this study, respectively. The design strength was set in accordance with KS F 4042 [16]. Through a preliminary mixing experiment, the water/binder ratio (W/M(B + S)) was determined to be 16%. Experiments were conducted by adding 0%, 1%, 3%, and 5% calcium nitrite relative to the weight of cement. Ordinary tap water and CO_2_ nanobubble water were used as mixing water, and eight types of mortar specimens were prepared.

The table flow, compressive strength, flexural strength, length change rate, and carbonation depth were measured. For microstructure analysis, the porosity was analyzed, and scanning electron microscopy (SEM) was conducted. The results of these experiments were used to examine whether the polymer cement mortar used for repairing concrete structures satisfied the KS F 4042 quality criteria (Table 3) [16].

### 2.2. Materials and CO_2_ Nanobubble Water

#### 2.2.1. Materials

Polymer cement mortar, in which an expansive admixture and polyvinyl alcohol (PVA) fibers were premixed as repair/reinforcement materials, was used in the experiments. Ordinary tap water (temperature: 20 °C, no impurities) and CO_2_ nanobubble water (particle diameter ~50 nm, pH adjusted to ~4.5 (slightly acidic)) produced using a nanobubble device (HACK FB11, Tokyo, Japan) were used as mixing water.

#### 2.2.2. Equipment and Process to Generate CO_2_ Nanobubble Water

**Figure 1:**
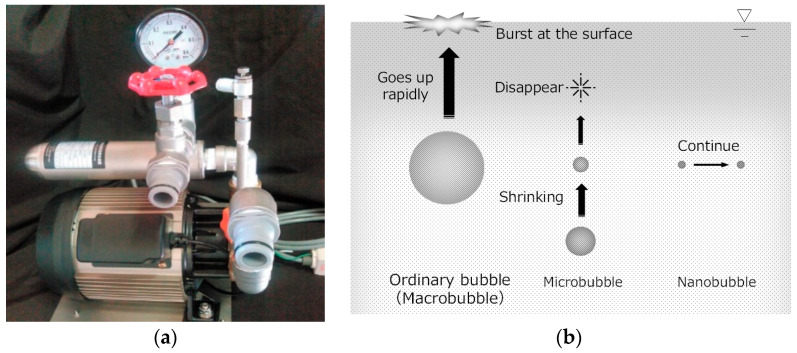
Equipment and process used to generate CO_2_ nanobubbles. (**a**) Depiction of the nanobubble device; (**b**) behavior of nanobubbles in water.

Figure 1 shows the equipment and process used to generate CO_2_ nanobubble water. A nanobubble generator connected to a CO_2_ source was operated in a water tank filled with tap water. The gas pump generated a negative pressure to pressurize the mixing header. CO_2_ saturation nanobubbles (particle diameter ~50 nm, pH adjusted to ~4.5 (slightly acidic)) generated by cavitation were then discharged through the foam nozzle into the water tank to produce CO_2_ nanobubble water [17,18]. These nanobubbles generally have small buoyancy and negative surface charges on the order of tens of microvolts; therefore, they repel and do not easily stick to each other, consequently lasting for a longer duration in water than microbubbles [19].

### 2.3. Experimental Parameters

In this study, the flow test was conducted to determine the workability of polymer cement mortar for repairing concrete structures. Physical properties such as strength, length change rate, and carbonation depth were tested to evaluate quality performances like densification and shrinkage behavior of the repair material, including calcium nitrite and CO_2_ nanobubbles as a mixing water. The porosity was analyzed to clarify the cause of densification through the change of the pore volume in each case over time. In addition, SEM analysis was performed to clarify the type and size of hydration products caused by the densification of the structure.

#### 2.3.1. Flow

For the flow of mortar (a non-hardening property) the experimental flow table of KS L 5111 [20] was used. The flow was measured immediately as well as 30 and 60 min after mixing in proportion with KS F 2476 [21].

#### 2.3.2. Compressive and Flexural Strength

**Figure 2:**
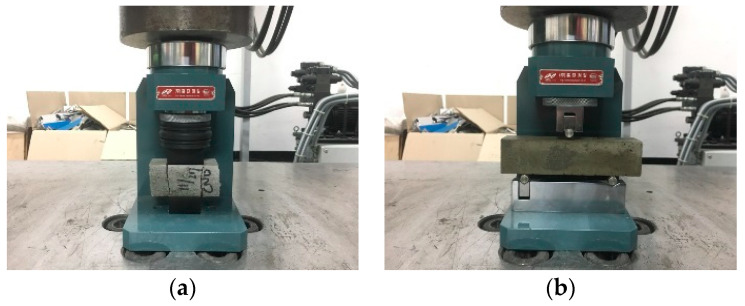
Pictures of the strength tests, with (**a**) the compressive strength test and (**b**) the flexural strength test.

Prismatic mortar specimens with a size of 40 × 40 × 160 mm were fabricated for the strength test in accordance with KS F 4042 [16]. They were demolded at two days of age and subjected to standard water curing at 20 °C. Their flexural strength was measured at 3, 7, and 28 days of age by using the central loading method and a universal testing machine (UH-F1000 kNX, Shimadzu, Kyoto, Japan) in accordance with KS F 2408 [22]. To test the compressive strength, specimens that were fractured after measuring their flexural strength were used. After mounting the specimens in a compression mold (40 × 40 × 40 mm), the compressive strength was measured at 3, 7, and 28 days of age in accordance with KS F 2405 [23]. Figure 2 shows the setup for measuring the compressive and flexural strengths.

#### 2.3.3. Length Change Rate

Prismatic mortar specimens (40 × 40 × 160 mm) in accordance with KS F 4042 [16] were used to test the length change rate. The specimens were demolded at two days of age and subjected to water curing at (20 ± 3) °C for five days. They were then cured in a constant temperature and humidity chamber at (20 ± 3) °C and (60 ± 5)% RH. The length change rate was measured for predetermined ages by using the dial gauge method of KS F 2424 [24].

#### 2.3.4. Carbonation Depth

The carbonation depth was measured by the accelerated carbonation test of KS F 2596 [25]. The acceleration conditions were at a temperature of (20 ± 2) °C, a humidity of (60 ± 5)%, and a CO_2_ concentration of (5 ± 0.2)%. The specimens were sealed with aluminum tape except at the top surface to block the infiltration of CO_2_. A solution of 1% phenolphthalein was sprayed on the split specimens, and the depth of the part that turned red was measured at 10 mm intervals by using a Vernier caliper.

#### 2.3.5. Porosity and SEM Analysis

Porosity measurements and SEM analysis were conducted only for specimens with 0% and 5% calcium nitrite to investigate the properties of tap water and CO_2_ nanobubble water according to the calcium nitrite content.

**Figure 3:**
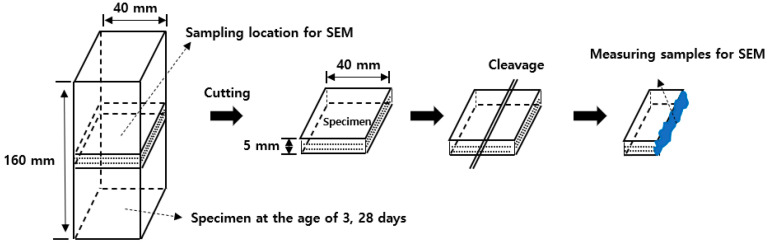
Specimen sampling for SEM.

As shown in Figure 3, samples were collected from the specimens fractured after measuring the strength at 3 and 28 days of age. The collected samples were used in the experiment after being immersed in acetone for more than four hours to stop the hydration reaction.

The samples used for porosity measurements were oven-dried at 60 °C. Mercury was then pressurized at 0–60,000 psi by using a porosity analyzer (AutoPore IV 9520, Norcross, GA, USA), and the pore size and cumulative pore volume of each specimen were measured from the amount of penetration. SEM analysis was conducted using the SNE-3200M device (SEC, Suwon, Korea), and the collected powder-type samples were coated with platinum. They were then observed at 3000× magnification at an acceleration voltage of 15 kV.

## 3. Results and Discussion

### 3.1. Flow

**Figure 4:**
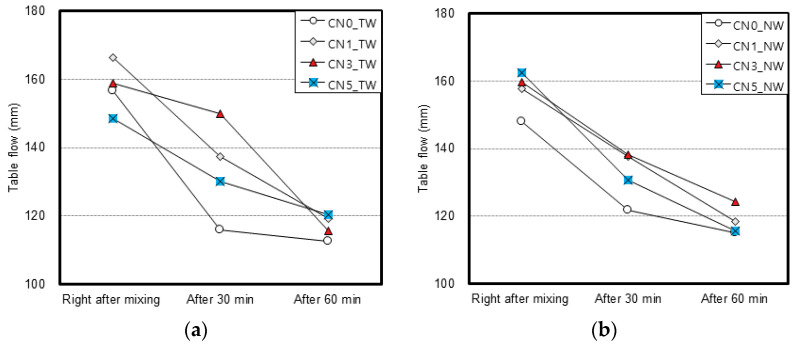
Flow test results: (**a**) result with tap water; (**b**) result with nanobubble water.

Figure 4 shows the flow test results. Regardless of whether tap water or CO_2_ nanobubble water was used, specimens with calcium nitrite tended to show high flow values. The flow did not seem to show any relation to the type of mixing water or the calcium nitrite content.

### 3.2. Compressive and Flexural Strength

**Figure 5:**
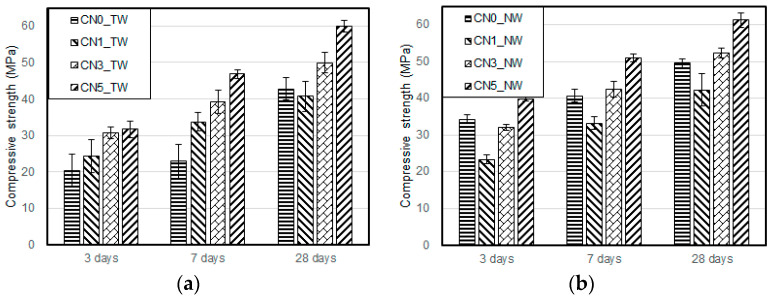
Compressive strength test results: (**a**) result with tap water; (**b**) result with nanobubble water.

**Figure 6:**
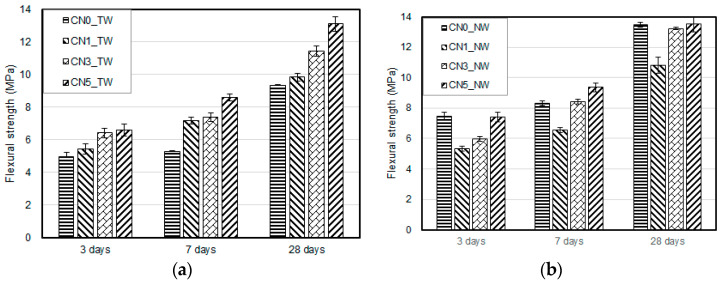
Flexural strength test results: (**a**) result with tap water; (**b**) result with nanobubble water.

Figure 5 and Figure 6 show the compressive and flexural strength results of the specimens when using tap water and CO_2_ nanobubble water according to the calcium nitrite content and age. For both types of mixing water, the compressive and flexural strengths increased with an increase in the calcium nitrite content. This was because NO_2_^−^ ions in calcium nitrite rapidly reacted with C_3_A(Al_2_O_3_), a component of cement, to increase the generation rate and amount of nitrite-based hydration products [26]. The CN0 specimen with CO_2_ nanobubble water showed somewhat higher compressive and flexural strengths because this water was initially produced at 30 °C, which was higher than the temperature for other specimens.

A comparison of the compressive and flexural strength results with the use of tap water and CO_2_ nanobubble water revealed a larger strength improvement effect with the latter. This result was similar to a previously reported result on the compressive strength of cement mortar using CO_2_ nanobubble water [27]. It seemed attributable to the increased generation of calcite-based CaCO_3_ in the cement matrix, as a large amount of Ca^2+^ ions was generated through the aforementioned accelerated the reaction between calcium nitrite, and cement reacted with the CO_3_^2−^ ions in the CO_2_ nanobubble water. This led to strength improvement via the densification of the structure [28,29,30].

For calcium nitrite content of 3% or higher, both the compressive and the flexural strengths satisfied the quality criteria of 20 and 6 MPa, respectively, specified in KS F 4042 [16] regarding the compressive and flexural strengths of polymer cement mortar required to repair concrete structures.

This indicated that a certain strength improvement effect can be expected if the CO_2_ nanobubble water and calcium nitrite contents are properly adjusted in consideration of site conditions at the time of the section restoration.

### 3.3. Length Change Rate

**Figure 7:**
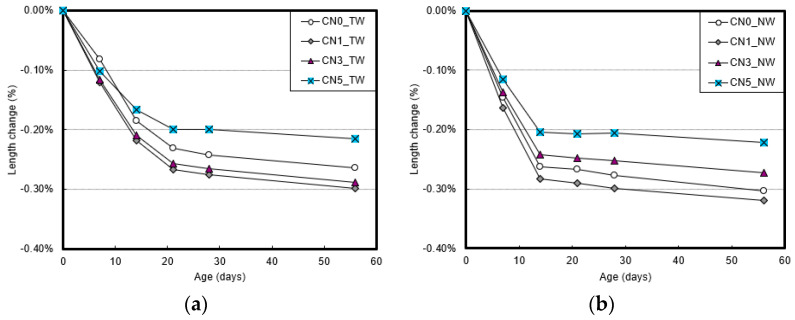
Length change rate of mortar: (**a**) result with tap water; (**b**) result with nanobubble water.

Figure 7 shows the length change rate measurement results of specimens that used tap water and CO_2_ nanobubble water according to the calcium nitrite content and age. As the age increased, the shrinkage also increased owing to drying and the rapid hydration reaction at early ages.

For tap water, the length change rate was higher with calcium nitrite contents of 1% or 3% than with contents of 0% or 5%. For CO_2_ nanobubble water, the length change rate decreased with calcium nitrite contents of 3% or higher. In particular, for calcium nitrite contents of 5% or higher, the length change rate decreased sharply regardless of the type of mixing water used. A shrinkage reduction effect of 0.05–0.08% was confirmed at 56 days of age compared to the specimen without calcium nitrite.

### 3.4. Carbonation Depth

**Figure 8:**
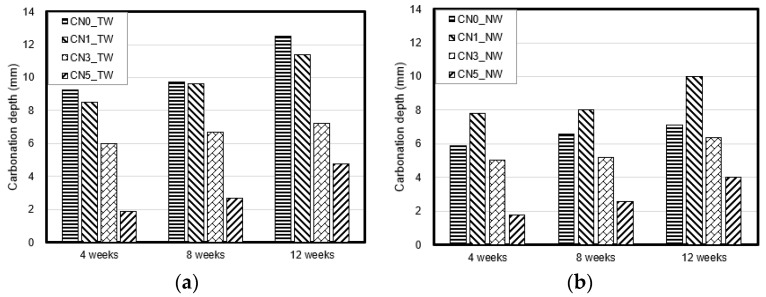
Carbonation depth: (**a**) result with tap water; (**b**) result with nanobubble water.

Figure 8 shows the carbonation depth measurement results of specimens that used tap water and CO_2_ nanobubble water according to the calcium nitrite content. With both types of mixing water, the carbonation depth decreased as the calcium nitrite content increased.

As mentioned in Section 3.2, this appears to be because the internal structure became denser with increasing calcium nitrite content as nitrite-based hydration products increased and, in particular, a large amount of ettringite was generated owing to the accelerated reaction between C_3_A(Al_2_O_3_) in cement and the NO_2_^−^ ions in calcium nitrite. In particular, when CO_2_ nanobubble water was used, an accelerated reaction occurred between the CO_3_^2−^ ions in CO_2_ nanobubble water and the Ca^+^ ions in cement. Therefore, the supply of CO_3_^2−^ ions alone caused the carbonation reaction, thereby increasing the generation of CaCO_3_ in the specimen. As a result, CO_2_ nanobubble water has a higher carbonation suppression effect owing to the densification of the structure [28,29,30]. In particular, when 5% calcium nitrite was added, the quality criterion of KS F 4042 [16] for the carbonation depth of polymer cement mortar for repairing concrete structures (less than 2 mm carbonation depth at four weeks of age) could be met.

This indicates that carbonation can be suppressed through the proper use of CO_2_ nanobubble water and calcium nitrite. Both are judged to be helpful in improving the performance of the section-restoration mortar via the densification of the structure of the cement matrix.

### 3.5. Porosity

**Figure 9:**
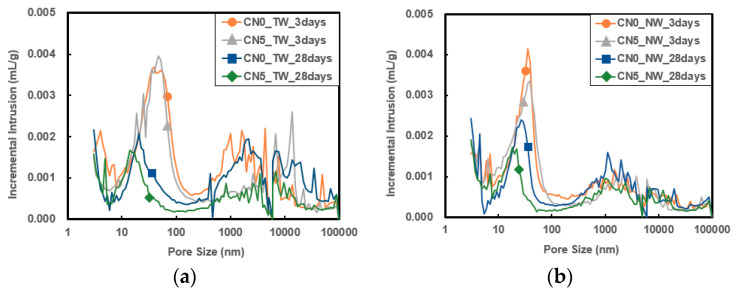
Incremental intrusion results: (**a**) result with tap water; (**b**) result with nanobubble water.

**Figure 10:**
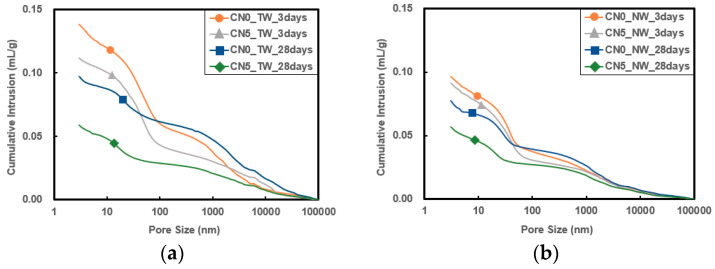
Cumulative intrusion results: (**a**) result with tap water; (**b**) result with nanobubble water.

Figure 9 and Figure 10 show the pore size distribution and cumulative pore volume results of specimens that used tap water and CO_2_ nanobubble water according to the calcium nitrite content and age. As the age increased, the cumulative pore volume decreased, indicating that the specimen structure became denser, thereby affecting the strength development. Overall, the addition of calcium nitrite slightly reduced the pore size and significantly reduced the cumulative pore volume.

In particular, when CO_2_ nanobubble water was used, the cumulative pore volume was smaller than with the use of tap water. This effect increased when calcium nitrite was used. As with strength development, this appears to be because the structure became denser through the accelerated reaction between C_3_A(Al_2_O_3_) in cement and the NO_2_^−^ ions in calcium nitrite as well as the reaction between the CO_3_^2−^ ions in CO_2_ nanobubble water and the Ca^+^ ions in cement. In addition, it is judged as a result of reducing the pore volume and size due to the formation of carbonate in the pores by the reaction between the water and the CO_3_^2−^ ions inside the pores [31,32,33].

### 3.6. SEM

**Figure 11:**
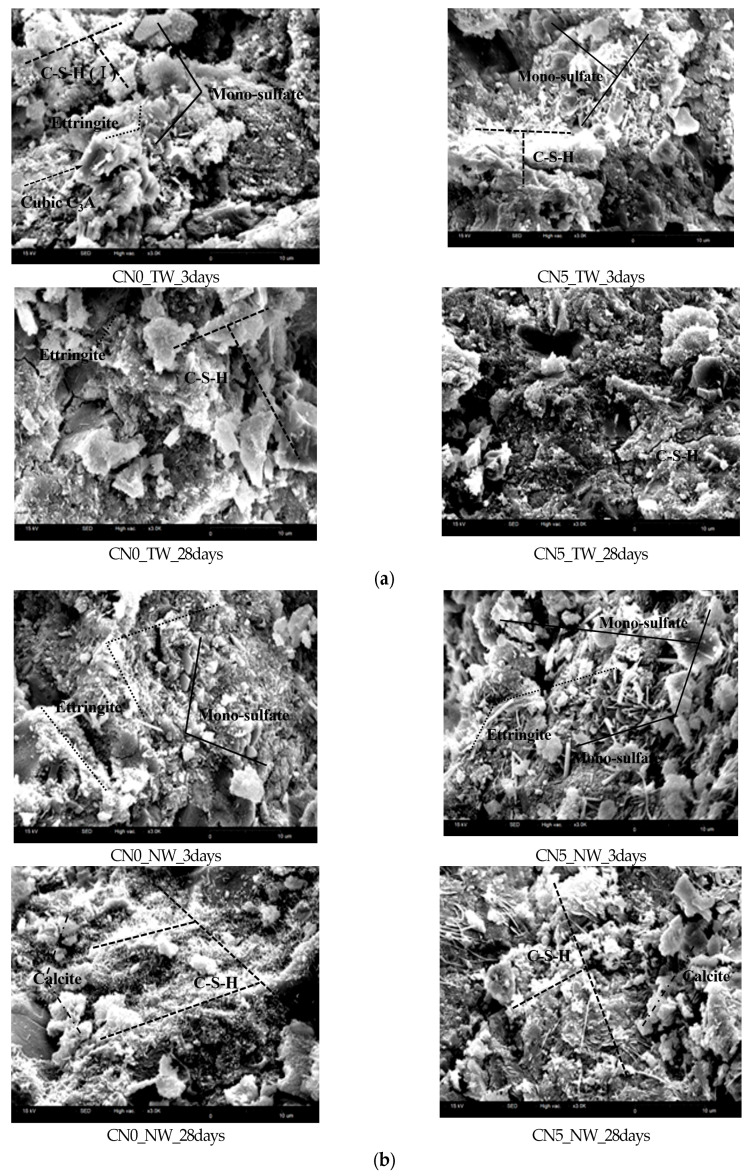
SEM (3000 × magnification): (**a**) result with tap water; (**b**) result with nanobubble water.

Figure 11 shows the SEM observation results of specimens that used tap water and CO_2_ nanobubble water according to the calcium nitrite content. Each hydration product was estimated by comparing the crystal structures obtained in this study with the crystal forms and sizes of hydration products confirmed in previous studies.

As the age increased, a wider distribution of hydration products, such as C-S-H gel and Ca(OH)_2_, was observed. In the case of the specimens without calcium nitrite, ettringite with its acicular brittle fracture behavior was distributed with monosulfate at an early age. In the case of the specimens with calcium nitrite, monosulfate was partially observed along with sulfuric acid (SO_4_^2−^)-based ettringite on the C_3_A surface of cement. Meanwhile, calcite was partially observed in the specimens that used CO_2_ nanobubble water containing CO_3_^2−^ ions. It appeared that CaCO_3_ (mainly calcite) was observed because the addition of calcium nitrite increased the generation rate of C-S-H gel and Ca(OH)_2_, and the CO_3_^2−^ ions in CO_2_ nanobubble water were adsorbed on the Ca^2+^ ions in the generated hydration products. In particular, nitrite-based hydration products and calcite-based CaCO_3_ were generated in large quantities in the specimens that used calcium nitrite and CO_2_ nanobubble water, indicating that the physical performance was improved through the densification of the structure.

## 4. Conclusions

In this study, the physical properties of section-restoration mortar using calcium nitrite and CO_2_ nanobubble water as mixing water were investigated. The following conclusions were derived:

1. The flow values of the specimens with calcium nitrite tended to be high. No special tendency according to the type of mixing water and the calcium nitrite content was seen. When 5% calcium nitrite was added, the length change rate sharply decreased.
2. As the calcium nitrite content increased, strength and durability also increased. In particular, the use of CO_2_ nanobubble water effectively increased the strength and reduced the carbonation depth and porosity.
3. As the calcium nitrite content increased, the generation rate and generated amount of nitrite-based hydration products increased owing to the rapid reaction between the NO_2_^−^ ions in calcium nitrite and the C_3_A(Al_2_O_3_) in cement.
4. A large amount of Ca^2+^ ions from Ca(OH)_2_ and C-S-H gel, which were generated through the accelerated reaction between calcium nitrite and cement, reacted with the CO_3_^2−^ ions in the CO_2_ nanobubble water, thereby increasing the generation of calcite-based CaCO_3_ in the cement matrix. This appears to have affected the strength development and durability improvement via the densification of the structure. The densification of the matrix appears to reduce the pore volume and affect strength development as well as durability improvement.

These results suggest that the performance of polymer cement mortar for repairing concrete structures can be improved if calcium nitrite and CO_2_ nanobubble water are properly combined and applied.

## Figures and Tables

**Table 1:** Experimental plan.

Item	Values	
Experimental variables and level	W/M (%)	16
	Ca(NO_2_)_2_ dosage ^1^	CN0 *, CN1, CN3, CN5 *
	Mixing water type	Tap water (TW), Nanobubble water (NW)
Evaluation items	− Flow − Compressive strength (3, 7, 28 days) − Flexural strength (3, 7, 28 days) − Length change − Carbonation depth (4, 8, 12 days) − Porosity * − Scanning electron microscopy micrograph (SEM) *	

^1^ CNx: x = Amount of Ca(NO_2_)_2_ (Binder × wt%). * Porosity and SEM analysis were conducted only for CN0 and CN5.

**Table 2:** Mix proportion of mortar.

W/M ^1^ (%)	B:S ^2^	Binder (wt%)						
		Cement	CSA	Resin	Anhydrous Gypsum	PVA Fiber	Superplasticizer	Viscosity Agent
16	1:1.45	89.5	6.6	1.5	1.2	0.6	0.58	0.04

^1^ M: = B + S, ^2^ B: Binder, S: Sand.

**Table 3:** Quality criteria (KS F 4042 [16]).

Evaluation Items	Quality Criteria
Compressive strength (N/mm^2^)	More than 6.0
Flexural strength (N/mm^2^)	More than 20.0
Carbonation depth (mm)	More than 2.0
